# Modular architecture of protein structures and allosteric communications: potential implications for signaling proteins and regulatory linkages

**DOI:** 10.1186/gb-2007-8-5-r92

**Published:** 2007-05-25

**Authors:** Antonio del Sol, Marcos J Araúzo-Bravo, Dolors Amoros, Ruth Nussinov

**Affiliations:** 1 Bioinformatics Research Unit, Research and Development Division, Fujirebio Inc., Komiya-cho, Hachioji-shi, Tokyo 192-0031, Japan; 2 Basic Research Program, SAIC-Frederick, Inc., Center for Cancer Research, Nanobiology Program, National Cancer Institute, Frederick, MD 21702, USA; 3 Sackler Institute of Molecular Medicine, Department of Human Genetics and Molecular Medicine, Tel Aviv University, Tel Aviv 69978, Israel

## Abstract

**Background:**

Allosteric communications are vital for cellular signaling. Here we explore a relationship between protein architectural organization and shortcuts in signaling pathways.

**Results:**

We show that protein domains consist of modules interconnected by residues that mediate signaling through the shortest pathways. These mediating residues tend to be located at the inter-modular boundaries, which are more rigid and display a larger number of long-range interactions than intra-modular regions. The inter-modular boundaries contain most of the residues centrally conserved in the protein fold, which may be crucial for information transfer between amino acids. Our approach to modular decomposition relies on a representation of protein structures as residue-interacting networks, and removal of the most central residue contacts, which are assumed to be crucial for allosteric communications. The modular decomposition of 100 multi-domain protein structures indicates that modules constitute the building blocks of domains. The analysis of 13 allosteric proteins revealed that modules characterize experimentally identified functional regions. Based on the study of an additional functionally annotated dataset of 115 proteins, we propose that high-modularity modules include functional sites and are the basic functional units. We provide examples (the Gα_*s *_subunit and P450 cytochromes) to illustrate that the modular architecture of active sites is linked to their functional specialization.

**Conclusion:**

Our method decomposes protein structures into modules, allowing the study of signal transmission between functional sites. A modular configuration might be advantageous: it allows signaling proteins to expand their regulatory linkages and may elicit a broader range of control mechanisms either via modular combinations or through modulation of inter-modular linkages.

## Abstract 2

A new method for studying signal transmission between functional sites by decomposing protein structures into modules demonstrates that protein domains consist of modules interconnected by residues that mediate signaling through the shortest pathways.

## Background

Allosteric communications play crucial roles in many cellular signaling processes. Perturbations caused by factors such as ligand binding at one functional site affect a distant site, thereby regulating binding affinity and catalytic activity [1,2]. Since the allosteric model proposed by Monod and coworkers [1], decades of research have extended the common view of allostery associated with multi-domain proteins to single domain proteins. The allosteric behavior displayed by single domain proteins, such as myoglobin [3], called into question the existing allosteric dogma. In the 'new view' of protein allostery, all proteins are potentially allosteric when thought of in terms of population redistribution upon ligand binding causing conformational change in a second binding site [1].

Dynamic models have been proposed to explain the conformational changes involved in signal transmission between functional sites [4,5]. In particular, the role of the pre-existing equilibrium of conformational sub-states in allostery proposed already over 20 years ago [6] is increasingly receiving attention, emphasizing the key role of protein dynamics in this process [1,7-9]. Although experimental methods such as double mutant cycle analysis [10] have provided insights into allosteric communications, understanding the general principles of the transmission of information between distant functional surfaces remains a challenge in structural biology. Several theoretical methods based on sequence and structural considerations have been proposed for the identification of key amino acids for long-range communications [11-13]. Among these, an interesting sequence-based approach has been proposed by Ranganathan and coworkers [14,15] for estimating the thermodynamic coupling between amino acids in several examples of protein families. Recently, we introduced a model based on a network representation of protein structures. The model allows us to determine fold centrally conserved residues (FCCRs). These residues are responsible for maintaining the shortest pathways between all amino acids and, thus, play key roles in signal transmission [13]. Analysis of several protein families showed an agreement between our results and experimental data, illustrating the importance of protein topology in network communications. Perceiving protein structures as information processing networks, it is reasonable to assume that mutations of amino acids crucial for network communications could impair signal transmission.

The rationale for modular organization of proteins in allosteric behavior has been discussed previously [16-18]. Modular domains can act cooperatively, leading to new input (and output) relationships. The Src family proteins constitute a clear example of this modular architecture: these proteins contain amino-terminal SH3 and SH2 domains, which flank a kinase domain by intra-molecular SH3-binding and SH2-binding sites [16]. It is further known that modular functional units display certain degrees of functional specificity in a number of proteins. In several cases of protein-protein interactions, which are involved in cell signaling, some parts of the interacting interface participate in the information transfer, whereas other interacting regions appear to contribute solely to binding affinity [19]. Examples of proteins exhibiting this binding site modular configuration include Myosin, C5a receptor, and the protein kinase R activator PACT among others [19]. Here, we aim to obtain the modular decomposition of allosteric proteins and to explore a relationship between the modules and the allosteric activity. We expect that such a relationship, if it exists, would lead to deeper insight into functional mechanisms. We develop a new approach for decomposing protein structures into modules using their residue network representations. Our methodology is based on the edge-betweenness clustering algorithm proposed by Newman and Girvan [20,21], which has been previously applied to a wide variety of problems [22-25]. This method uses edge centrality to detect module boundaries and finds the assignation of nodes into modules [20].

The small-world topology of protein structures suggests that the key amino acids for signal transmission should lie in the shortcuts linking different regions of the structure. The removal of the most central contacts forming these shortcuts divides the structure into modules. We characterize these modules from a structural point of view. Our results, derived from a non-redundant dataset of multi-domain proteins, reveal that, in the vast majority of the cases, modules tend to be located within rather than across domains. Therefore, modules can be considered as sub-domains. Further analysis shows that the percentage of long-range interactions at the modular boundaries is much higher than that in non-boundary regions. Residues forming inter-modular contacts fluctuate less than those participating only in the intra-modular interactions. One possible explanation of this finding is that most central residues, which have been shown to be important for the allosteric communications, are located at the inter-modular interfaces and, therefore, tend to be more rigid to maintain their contacts. Inspection of 13 allosteric proteins shows that functionally annotated regions exhibit a modular architecture, with modules interconnected by FCCRs, which are responsible for mediating the shortest pathways between all amino acids and, thus, play crucial roles in allosteric communications [13]. Functional sites are often contained in one module; however, there are also examples of functional sites shared by two or more modules. Some of these cases correspond to binding sites divided into two modules belonging to different domains. The Gα_*s *_subunit and P450 cytochromes are examples of functional sites shared between modules. Interestingly, the modular decomposition of the Gα_*s *_subunit reflects binding site partitioning into regions involved in different sub-functional specialization, general binding and information transfer regions [26]. The P450eryF active site is divided into a module containing the ligand-binding site, and a module comprising the effector-binding site, whereas the P450cam substrate binds to one module, and the product binds mainly to another module. A detailed analysis of a large dataset of proteins with functional annotations revealed that modules exhibiting high modularity tend to include functional sites.

Our results lead us to propose that the modular architecture of protein structures yields a more efficient performance of the functional activity. Modules may possess certain functional independence; and, they are interconnected through amino acids previously shown to mediate signaling in proteins. Modules consist of groups of highly cooperative residues. Evolution has organized proteins as systems consisting of modules linked by amino acids that maintain the shortest pathways between all amino acids and are, thus, crucial for signal transmission, leading to robust and efficient communication networks. This organization is advantageous and, as such, has been conserved by evolution.

## Results and discussion

Here we propose a novel way to decompose protein structures into modules based on their representation as residue interacting networks (see Materials and methods). Our approach relies on the edge-betweenness clustering algorithm presented by Newman and Girvan [20,21]. Modular decomposition allows us to identify functionally important regions in proteins.

### Structural properties of modules

**Figure 1:**
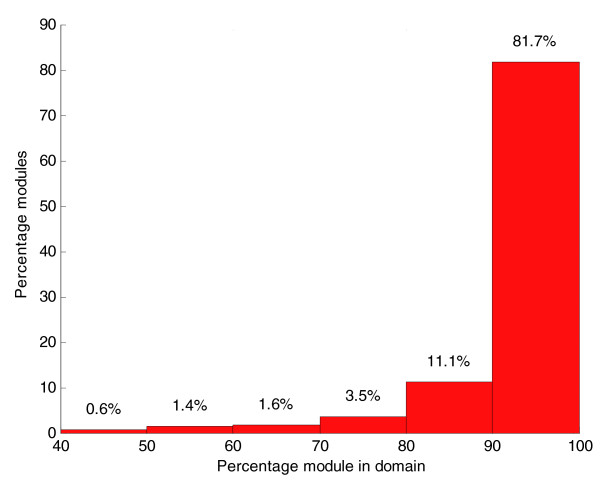
Mapping of modules into domains for the dataset of multi-domain proteins. The abscissa axis shows the percentage of a module contained in one domain. The bars indicate the percentage of all modules corresponding to each interval of the abscissa axis.

**Figure 2:**
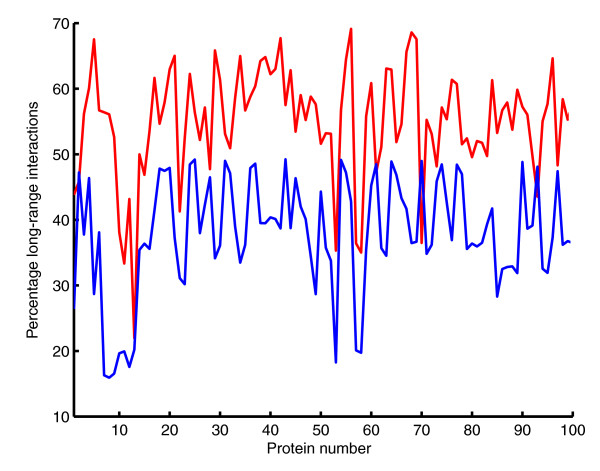
Percentage of long-range interactions for each protein of the multi-domain protein dataset. The interactions were calculated separately for the set of the inter-modular residues and for the set of intra-modular residues. The ordinate axis shows the percentage of long-range interactions for the inter-modular interfaces (in red) and for the intra-modular regions (in blue).

We carried out the modular decomposition of protein structures of a non-redundant dataset of 100 multi-domain proteins (described in Materials and methods). Results show that the majority of the modules have most of their residues in one domain (Figure 1). That is, modules tend to be located within rather than across domains, and hence may be considered as sub-domains. Comparison of contacts between amino acids belonging to different modules (inter-modular contacts) and those between amino acids belonging to the same module (intra-modular contacts) revealed that the percentage of long-range interactions is larger in the inter-modular contacts (Figure 2). This finding is in agreement with the rationale that long-range interactions often mediate the shortest pathways between most residues in the protein.

**Figure 3:**
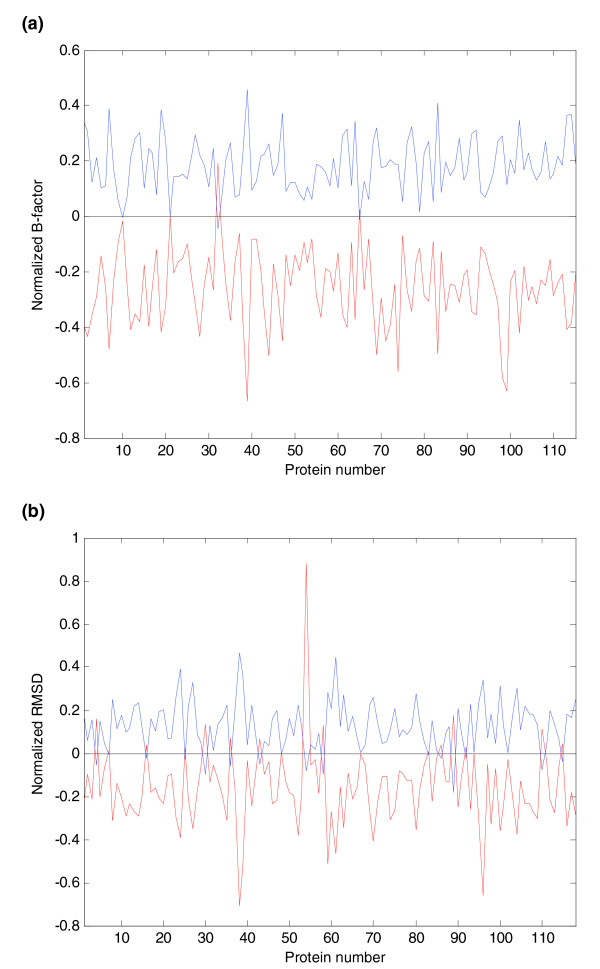
Modular flexibility for each protein of the dataset of proteins with conformers. **(a) **Averages of normalized residue temperature B-factors for inter-modular residues (red) and intra-modular residues (blue) for each protein. **(b) **Averages of normalized residue RMSDs for inter-modular residues (red) and intra-modular residues (blue) for each protein.

A detailed analysis of 115 proteins (described in Materials and methods) with available structures in different conformational states and temperature B-factors showed that residues with inter-modular contacts fluctuate less than those forming exclusively intra-modular contacts. Figure 3 clearly illustrates this situation: the normalized root mean square deviation (RMSD) values and the B-factors of the residues involved in inter-modular interactions tend to be lowerthan those of the residues involved in intra-modular interactions. This result could suggest that intra-modular regions, which include most of the protein or ligand binding sites, absorb conformational changes due to perturbations. In contrast, the boundaries between modules are more rigid, allowing them to maintain key residue contacts for the integration and transmission of the information between modules.

### Modularity of protein function

The modular decomposition of protein structures provides information about functional sites and signal transmission. We selected a dataset of 13 allosteric proteins based on previously analyzed examples [13] and new examples with experimental information. A detailed study of these proteins revealed that many modules contain functional regions, which are interconnected by residues mediating the shortest pathways between most amino acids in the structure (FCCRs). A majority (72%) of the FCCRs connect modules (Additional data file 1). Table 1 summarizes the analyzed examples, including the assignment of functional sites to modules (detailed information is provided in Table 3 of Additional data file 1).

**Table 1:** Modular division and FCCRs connecting functional modules for the studied allosteric proteins

Protein (ID)	Functional sites	Modules	Linking FCCRs
Hemoglobin (1bz0 A) ['15,48,49]	Hem BS	1,2	65(1)(1-2)
	AB interface	1	66(1)(1-2)
			98(2)(2-1)
			128(1)(1-2)
Glycogen phosphorylase (1e1y A) ['50-53]	Cat site	5	84(2)(2-3-4)
	AMP BS	1	93(1)(1-2-5)
	280 loop*	2	138(2)(2-1-4)
	Glycogen BS	2	161(2)(2-1)
	Tower helix*	2	490(1)(1-2-5)
			608(5)(5-2)
			648(5)(5-2-4)
Retinoic acid receptor RXR-alpha (1g5y A)	Cat ligand BS	5	305(1)(1-5)
[54-56]	AF2 helix*	4	309(5)(5-3-1)
	Coactivator BS	1,4	310(5)(5-1)
	AB interface	5	315(5)(5-1)
			371(1)(1-5)
Catabolite gene activator protein (1g6n A)	DNA BS	3	63(2)(2-3)
[57-61]	cAMP BS	2,1	64(2)(2-1-4)
			65(2)(2-1-3)
			69(1)(1-2-4)
Glutamate dehydrogenase (1hwz A) [62-64]	Cat site	2	110(2) (2-1)
	NAPH BS	*Dom*A2 in 2 and *Dom*A3 in 5,1	173(2) (2-5)
	GTP BS	5	211(2) (2-5-1)
	Glutamate BS	*Dom*A2 in 2 and *Dom*A3 in 2	252(5) (5-1)
	Antenna*	7	347(1) (1-5)
Rhodopsine (1l9h A) [65-69]	Retinal BS G protein BS	1 2	301(1)(1-3-2)
Pyruvate kinase (1liu A) [70,71]	Cat site	*Dom*A2 in 5 and *Dom*A3 in 7	163(7)(7-4)
	FBP BS	3	337(6)(6-2-7)
	PEP BS	6,4	342(7)(7-6)
			361(6)(6-2-3)
			482(3)(3-6-2)
			488(3)(3-6-2)
Phosphofructokinase (1pfk A) [72-74]	Cat site	*Dom*A1 in 2 and *Dom*A2 in 3	126(2)(2-3)
	FBP BS	*Dom*A1 in 2 and *Dom*A2 in 3	139(2)(2-3)
	MgADP BS	2,1	169(3)(3-2)
Tyrosine phosphatase 1B (1pty) [28]	Cat site	2	81(2)(2-1)
	Phosphotyrosine BS	2,1	109(2)(2-1)
	Inhibitor BS	1	194(1)(1-2)
			199(1)(1-2)
			254(1)(1-2)
			257(2)(2-1)
Beta-trypsin (2ptc E) ['15,74,76-78]	Cat site	*Dom*A2 in 3 and *Dom*A1 in 3	29(3)(3-1)
	S1 site*	2,1	30(3)(3-1)
	Loop1*	2	138(1)(1-2-3)
	Loop2*	2	141(1)(1-3)
	Loop3*	2	189(2)(2-1)
			194(1)(1-3-2)
			212(3)(3-2)
			213(3)(3-1-2)
			228(2)(2-1-3)
G-protein s-alpha (1azs C) [19,26,29]	Cat site	*Dom*C1 in 1,4 and *Dom*C2 in 1	50(1)(1-4-3)
	GSP BS	*Dom*C1 in 1 and *Dom *C2 in 4	58(1)(1-4)
	Adenylyl cyclase BS* -Binding only*	*Dom*C2 in 4,1 and *Dom*C1 in 1 2	173(4)(4-1-5) 201(4)(4-1-3)
	-Binding and transmission*	*Dom*C2 in 4,1 and *Dom*C1 in 1	
G-protein beta-gamma (1tbg A) [19,79]	PLC-beta2 BS*	4	61(4)(4-3-2)
	-Binding only*	3,2,4	63(4)(4-3-2)
	-Binding and transmission*	4	105(4)(4-3)
			150(3)(3-4)
			151(3)(3-4)
			190(3)(3-2)
			192(3)(3-2)
			234(2)(2-3)
			258(2)(2-1-3)
			289(2)(2-4)
			318(2)(2-4)
			320(2)(2-4)
Cytochrome P450eryF (1eup A) [32]	Hem BS	2,6	102(6)(6-2-4)
	Andro1 BS	6	238(6)(6-3)
	Andro2 BS	3	349(2)(2-5-6)

The functional site divisions into modules are indicated. *The information on these sites was extracted from the reference indicated in the first column. *Dom *denotes those functional sites divided into several domains according to the CATH database. The FCCRs linking functional modules are listed (the first number in parentheses represents the module to which the FCCR belongs and the numbers in the following parentheses are the modules it connects). BS, binding site; Cat, catalytic site. AB, chains A and B; AF2 helix, activation function 2 helix; FBP, fructose1,6-bisphosphate; PEP, phosphoenolpyruvate; PLC, phospholipase C.

### Modular division of functional sites

Functional sites can be decomposed into modules. In some cases, the modules are located in different domains. An illustrative example of this situation is the pyruvate kinase (PDB ID 1liu, chain A). The catalytic site is divided into two modules belonging to different domains and exhibiting different degrees of flexibility [27] (Table 1). In other examples, the functional site is contained in one domain and is divided into two or more modules. Such is the case of tyrosine phosphatase 1B (PDB ID 1pty), with the catalytic residues located in two modules. One of these modules comprises a loop, whose flexibility is important for the transition from the open to the closed conformation [28] (Table 1). The Gα_*s *_subunit and Cytochrome P450eryF and P450cam examples are discussed in detail below.

#### Guanine nucleotide-binding protein G(s) subunit alpha (*Bos Taurus*)

**Figure 4:**
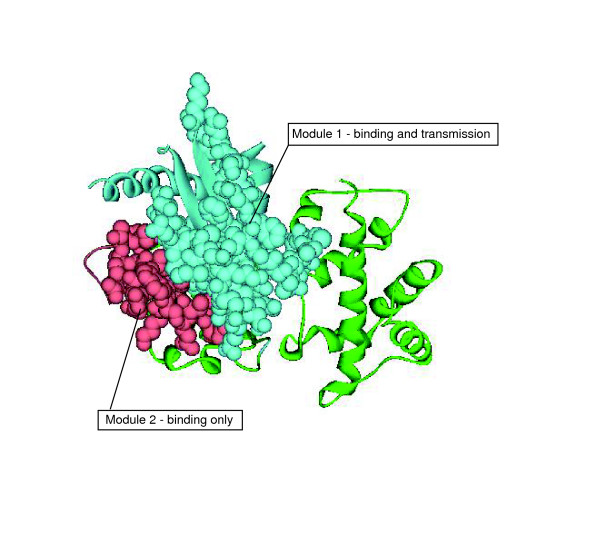
Binding site of the G-protein α s subunit (PDB ID 1azs) divided into two modules. This division coincides with the specialized regions of this binding site for ligand binding only (pink module) and ligand binding and information transfer (blue module). The binding site residues are depicted in spacefill. Modular regions not involved in the binding site are depicted in green.

A well-studied example of signal transmission is the regulation of adenylyl cyclase by the Gα_*s *_subunit [19,29]. It is known that the Gα_*s *_subunit undergoes significant conformational changes upon exchange of GDP by GTP, affecting its affinity for adenylyl cyclase [29]. It has been experimentally verified that the Gα_*s *_subunit involves three main regions for its interaction with this enzyme effector - the switch I and switch II regions and the α *3-β5 *loop [26]. Although the Gα_*s *_subunit activation of adenylyl cyclase is a complex process, experimental results indicate that the switch I and switch II regions, which display conformational flexibility, mainly mediate information transfer, whereas the α *3-β5 *loop is solely involved in the ligand binding affinity [26]. Interestingly, the modular decomposition of the Gα_*s *_subunit (1azs, chain C) shows that the adenylyl cyclase-binding site is divided into two modules: one of the modules contains the switch I and switch II regions and the other module comprises the α *3-β5 *loop (Figure 4). Thus, in this example we find a correspondence between the modular decomposition of the binding site and its partition into signal-transfer and general binding regions.

#### Cytochromes P450

##### P450eryF (*Saccharopolyspora erythraea*)

**Figure 5:**
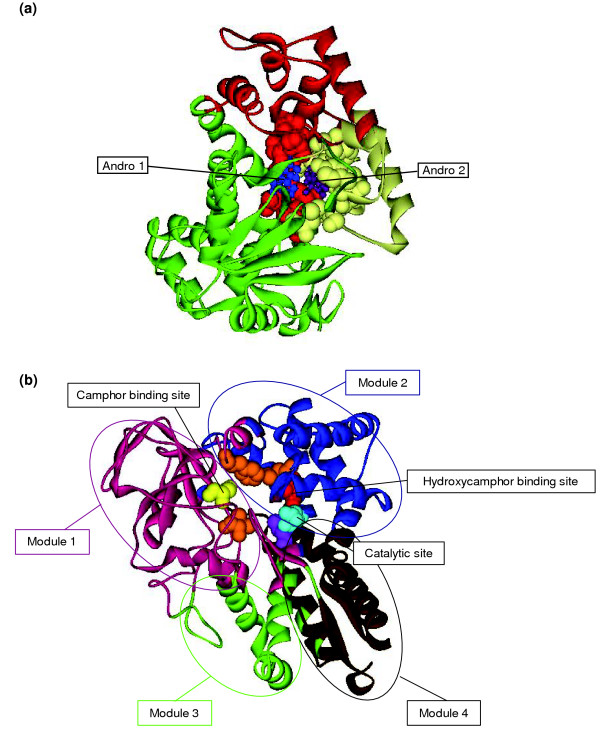
Modular division of the Cytochromes binding sites. **(a) **Modular division of the Cytochrome P450eryF (PDB ID 1eup) binding site. Two androstenedione molecules (Andro1 and Andro2 colored in blue and purple, respectively) are bound to the protein. The binding site (in spacefill) for the androstenedione is divided into two modules (highlighted in red and yellow) corresponding to the binding area for each of these two molecules. Modular regions not involved in the binding site are depicted in green. **(b) **Modular division of the Cytochrome P450cam (PDB ID 1noo) binding site. Two camphor molecules (camphor and 5-exo-OH camphor) can bind to the protein. The binding site (in spacefill) for the camphor is highlighted in yellow and orange. The binding site (in spacefill) for the 5-exo-OH camphor is highlighted in red and orange. Residues in orange are the ones that can bind both camphor and hydroxycamphor. Catalytic residues (in spacefill) are highlighted in light blue and purple. The ones in purple can also bind hydroxycamphor. The residues forming each of the four modular regions (and not involved in any of the functions previously described) are depicted in magenta, blue, green and brown.

P450eryF, a cytochrome P450 involved in erythromycin biosynthesis, exhibits no cooperativity with its natural substrate 6-deoxyerythronolide, while showing sigmoidal substrate saturation curves with other smaller substrates [30]. The presence of multiple binding sites within the same binding pocket is believed to be a primary cause of allostery in cytochromes P450 [31]. Since P450eryF has a large active site, it is assumed that P450eryF is capable of binding the large substrates of the mammalian P450s [32]. X-ray crystallographic studies and other experimental results indicate that two androstenediones are simultaneously present in the active site, interacting with each other, and, therefore, exhibiting a certain degree of homotropic cooperativity [32]. Binding of one androstenedion (Andro2) induces conformational changes in the active site and increases its hydrophobicity, resulting in increased binding affinity to the other androstenedion (Andro1) [32]. The modular decomposition of this protein indicates that the two modules share the active site. Each of these modules contains one of the two androstenedion-binding sites (Figure 5a).

##### P450cam (*Pseudomonas putida*)

The camphor monoxygenase P450cam catalyzes the 5-exo hydroxylation of camphor [33]. Its active site may be considered to have two functionally different subsites: the substrate binding region (site I) and the L_6 _position of the iron to which oxygen binds upon reduction (site II) [33]. Allosteric interactions between these subsites are reflected in the fact that site I binding can inhibit site II ligation and vice versa. Furthermore, the presence of the product 5-exo-OH camphor inhibits binding of the substrate camphor (and vice versa) [33]. The modular decomposition of the P450cam structure (PDB ID 1noo) shows that the substrate (camphor) and product (5-exo-OH camphor) binding sites are mainly located in different modules, sharing common central residues, which are likely to be important for the allosteric communication between these sites. Figure 5b shows that residues comprising the 5-exo-OH camphor binding site tend to be located closest to the heme central ion, whereas amino acids forming the camphor binding site tend to be positioned distal from the heme group.

These examples suggest that the modular design of functional sites might be related to their sub-functional specialization. Each module contains a portion of the active site and is mainly involved in a specific sub-function, such as the binding of the substrate, the product or an allosteric ligand.

### Modularity and functional significance of modules

**Figure 6:**
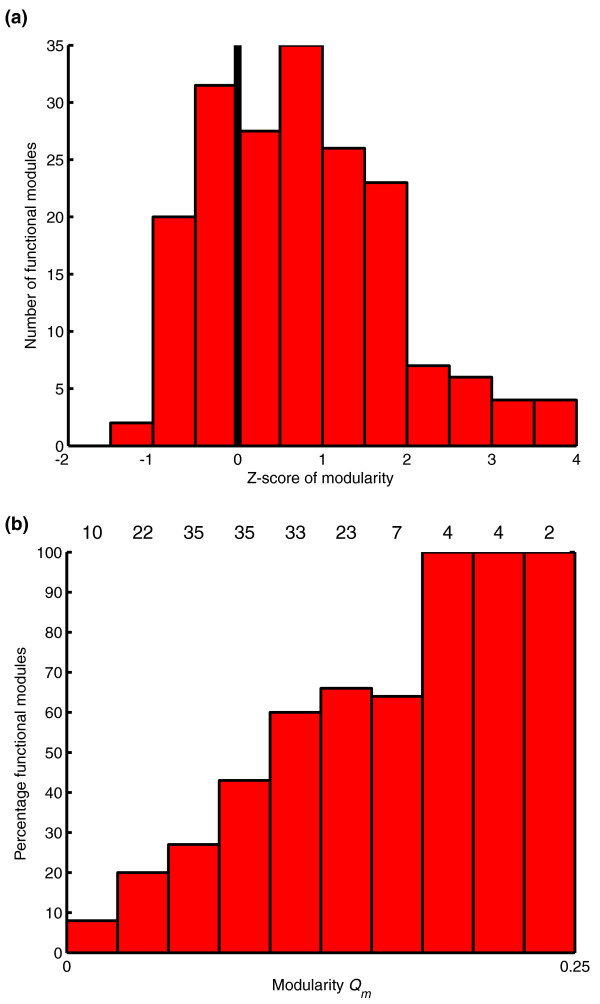
Relationship between functionally annotated modules and modularity. **(a) **Z-score distribution of the modularity values for functional modules. The abscissa axis represents the Z-score modularity values calculated for all modules. The vertical line at Z-score = 0 represents the averaged modularity of all modules. The bars stand for the number of functional modules for each Z-score interval shown in the abscissa. **(b) **Distribution of modularity values for functional modules. The abscissa axis shows the different intervals of modularity. The bars represent the percentage of functional modules for each interval of modularity. The number of functional modules for each range of modularity is indicated at the top of the graph.

Analysis of the previously studied dataset of 115 proteins with functional site annotations (described in Materials and methods) indicates that modules exhibiting high modularity values tend to comprise functional sites. The analysis of all modules illustrates that a large percentage of modules comprising functional regions exhibit above average modularity values (Figure 6a). Figure 6b clearly illustrates that there is a correlation between the percentages of functional modules and the modularity values.

## Conclusion

In signaling proteins, modular domains can act as switches mediating activation, repression and integration of diverse input functions. Experimental studies confirm that inter-domain linker regions are crucial for the domain coupling required for the information transfer [16]. Our approach decomposes protein structures into modules, allowing us to study functional sites linked by signal transmission. To detect module peripheries, we rely on the identification and removal of the most central residue contacts, assuming that the interactions of these amino acids are crucial for information transfer. Our results show that modules, which often characterize functional sites, can be considered as building blocks of protein domains. Hence, the question arises, how is the transmission between distinct modules achieved? Although a very complex process, which is not fully understood, our findings suggest that inter-modular boundaries are essential for integrating and transmitting the information between functional regions. The majority of the fold centrally conserved residues, recently shown to play a key role in signal transmission by maintaining the short path lengths between all residues in the structure [12], are those responsible for the inter-modular interactions. Furthermore, boundary residues are rigid, sustaining key amino acid interactions for the communication between modules. On the other hand, intra-modular regions, which include most of the protein or ligand binding sites, form a flexible cushion. Most of the inter-modular residue interactions form long-range contacts, which are predominantly involved in mediating signaling. A detailed study of 13 allosteric proteins showed that functional sites are often contained within one module. However, there are cases of active sites divided into two or more modules. The analysis of the Gα_*s *_subunit and of Cytochromes P450eryF and P450cam illustrate that the modular architecture of the active site may relate to its sub-functions. Modules containing functional sites display high modularity, suggesting that modularity can be used to identify functional modules.

To conclude, our approach decomposes protein domains into modules. Mapping annotated functional regions onto the decomposed structures illustrates that the modules characterize functional sites. We observe that most inter-modular boundary residues provide the shortcuts in the communication wires. These residues maintain the shortest pathways between all amino acids, leading to robust and efficient signal transmission communication networks. Functional specificity and regulation relies on the communication between modules. This advantageous organization has been conserved by evolution. Furthermore, due to the possible functional independence of modules, changes in boundary residues may lead to new functions or to functional alterations as might be needed in a changing environment. Therefore, a modular configuration might allow signaling proteins to increase their regulatory links, and to expand the range of control mechanisms either via new modular combinations or through modulation of inter-modular linkages. Since our results indicate that boundary residues are crucial in efficient short communication pathways, both mechanisms appear possible.

## Materials and methods

### Protein datasets

A non-redundant dataset of 100 multi-domain proteins was selected from NCBI [34]. The domain information was extracted from the CATH database [35,36]. This dataset was used to analyze the distribution of protein modules into domains and to calculate the distribution of the long-range interactions at the inter-modular interfaces and in the intra-modular regions. Using the definition of Green and Higman [37], we considered the interactions as long range if they occur between amino acid residues that are ten or more residues apart in the sequence. While residues close in sequence are close in space, we adopt this standard notation, which has been used in numerous studies. The analyses of flexibility and modularity of modules were based on a different dataset of 115 proteins with conformers. This dataset was compiled using the database of macromolecular movements: [38-40] undergoing distinct molecular motions. Only conformers with more than 60% sequence identity were chosen. The annotations of functional sites were taken from PDBsum [41,42]. We annotated a module as functional if more than 30% of its residues belong to a functional site. We selected 13 examples of proteins displaying allosteric activities with existing PDB structures. All protein structure images were created using DS ViewerPro 6.0 [43].

### Network analysis of protein structures

Each protein structure was modeled as an undirected graph, where amino acid residues corresponded to vertices, and their contacts were represented as edges. Residues i and j were considered to be in contact if at least one atom corresponding to residue i was at a distance of less than or equal to 5.0 Å from an atom from residue j. This value approximates the upper limit for attractive London-van-der-Waals forces [12,37].

FCCRs were calculated as in del Sol *et al*. [13]. Protein networks were decomposed into modules using the edge-betweenness clustering algorithm of Girvan and Newman [21] based on the iterative removal of the highest betweenness edges. We used the parallel implementation PEBC (parallel edge betweenness clustering) [44] of the Girvan and Newman algorithm. We modified the program to obtain the modular decomposition after removing 80% of the network edges. This cutoff was obtained empirically for optimizing the correspondence in the mapping of functional sites into modules. Based on the expression of network modularity introduced by Guimerà and Nunes Amaral [45], we defined the modularity of protein modules *Q*_*m *_as follows:

Qm=lmL−(dm2L)2
 MathType@MTEF@5@5@+=feaafiart1ev1aaatCvAUfeBSjuyZL2yd9gzLbvyNv2Caerbhv2BYDwAHbqedmvETj2BSbqee0evGueE0jxyaibaiKI8=vI8tuQ8FMI8Gi=hEeeu0xXdbba9frFj0=OqFfea0dXdd9vqai=hGuQ8kuc9pgc9s8qqaq=dirpe0xb9q8qiLsFr0=vr0=vr0dc8meaabaqaciGacaGaaeqabaqadeqadaaakeaacaWGrbWaaSbaaSqaaiaad2gaaeqaaOGaeyypa0ZaaSaaaeaacaWGSbWaaSbaaSqaaiaad2gaaeqaaaGcbaGaamitaaaacqGHsisldaqadaqaamaalaaabaGaamizamaaBaaaleaacaWGTbaabeaaaOqaaiaaikdacaWGmbaaaaGaayjkaiaawMcaamaaCaaaleqabaGaaGOmaaaaaaa@4038@

where *L *is the number of edges in the network, *l*_*m *_is the number of edges between nodes in module *m*, and *d*_*m *_is the sum of the degrees of the nodes in module *m*. The rationale for this modularity measure is as follows: modules with high modularity values must contain many within module links and as few as possible between-module links. The equation above imposes *Q*_*m *_= 0 in cases when the module comprises the whole network or if nodes are placed randomly into modules.

### Protein flexibility analysis

The analysis was carried out over the dataset of 115 proteins with conformers in two ways. We first calculated the averaged main chain residue RMSD considering all pairs of structurally aligned conformers. The structural alignments were obtained using MultiProt [46,47]. We also calculated the main chain temperature B-factor of each residue. The normalizations of the RMSDs and B-factors were calculated using the standard definition of the Z-score values.

## Additional data files

The following additional data are available with the online version of this paper. Additional data file 1 contains figures with additional examples of protein modularity and tables with the data sets used for the analyses.

## Supplementary Material

Additional data file 1 Additional examples of protein modularity and the datasets used for the analyses. Click here for file
