# Supplementary material for: Modular architecture of protein structures and allosteric communications: potential implications for signaling proteins and regulatory linkages
Source: Genome Biol. 2007 May 25;8(5):R92. doi: 10.1186/gb-2007-8-5-r92 (PMC1929157; doi:10.1186/gb-2007-8-5-r92)
Supplement: Additional data file 1 — Additional examples of protein modularity and the datasets used for the analyses. [file gb-2007-8-5-r92-S1.doc]

**Additional data files**


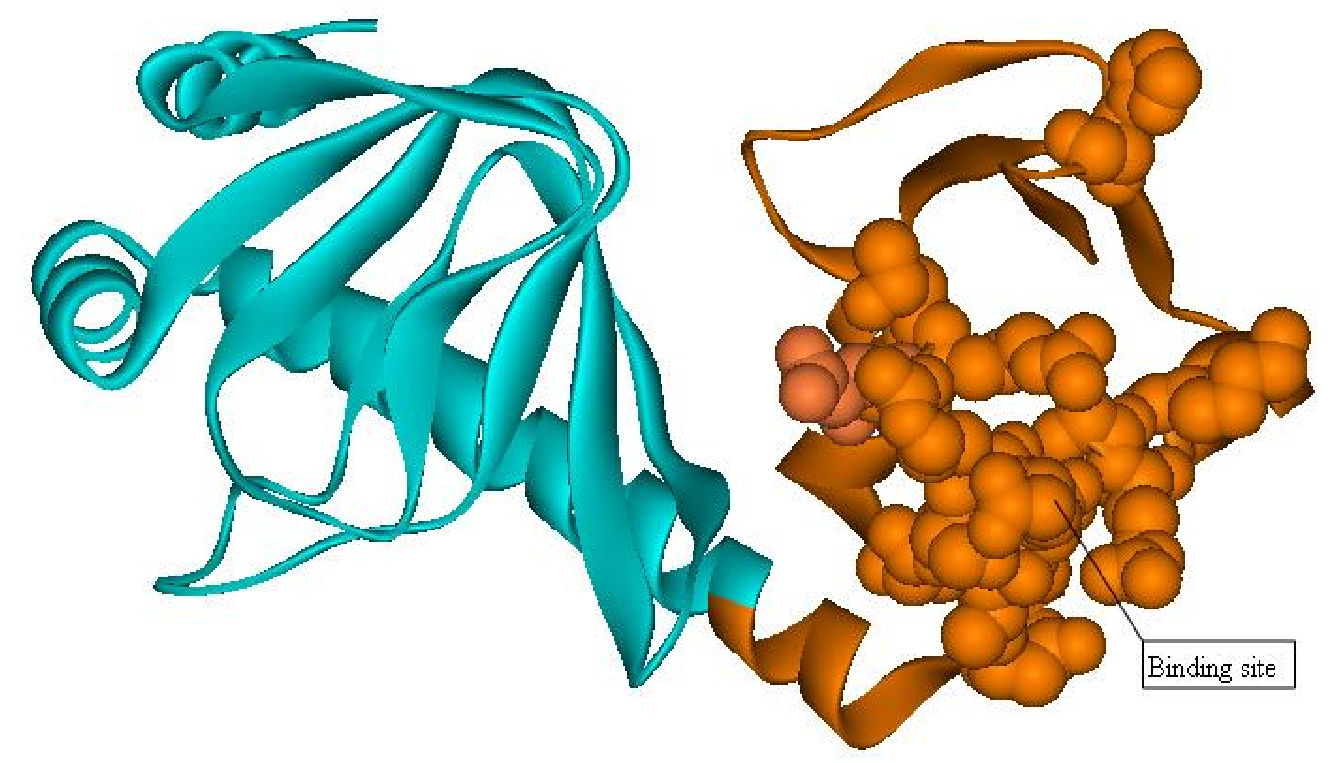


**Fig 1 Supp** Representation of one binding site included in one module on the structure of the CAP protein (PDB ID: 1g6n). The area colored in orange represents one of the modules of the protein. The DNA binding site (shown in spacefill) is located completely inside this module. Modular regions not involved in the binding site are depicted in cyan.


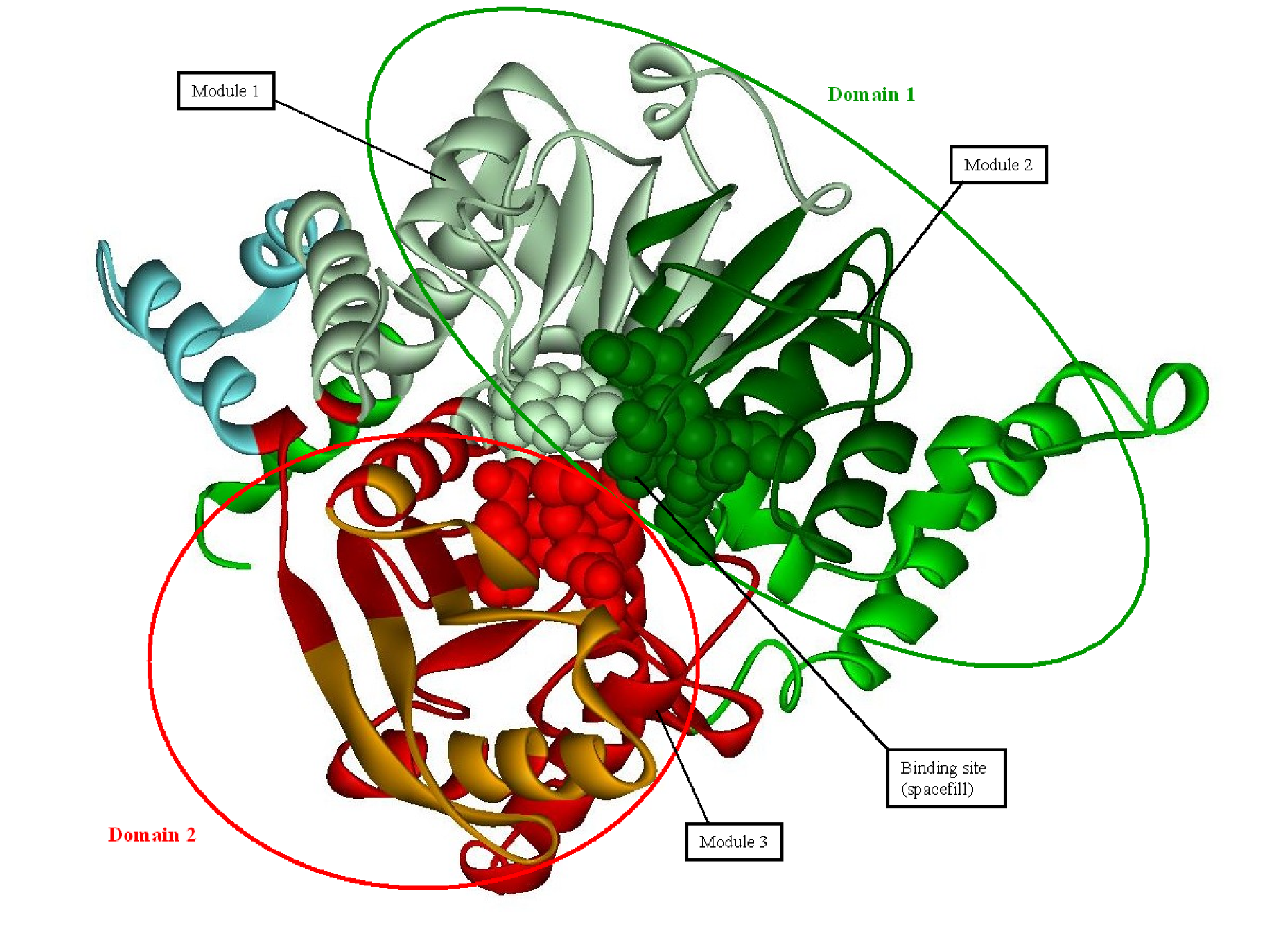


**Fig 2 Supp** Representation of the glutamate dehydrogenase (PDB ID: 1hwz) showing a binding site divided in two domains (framed by ovals) and three modules (labeled Module 1, 2 and 3). One domain is depicted in green range (formed by three modules, colored lightgreen, green and darkgreen) and the other one represented in red range (composed of two modules, one depicted in red and the other one in orange). The residues of the binding site are shown in spacefill.


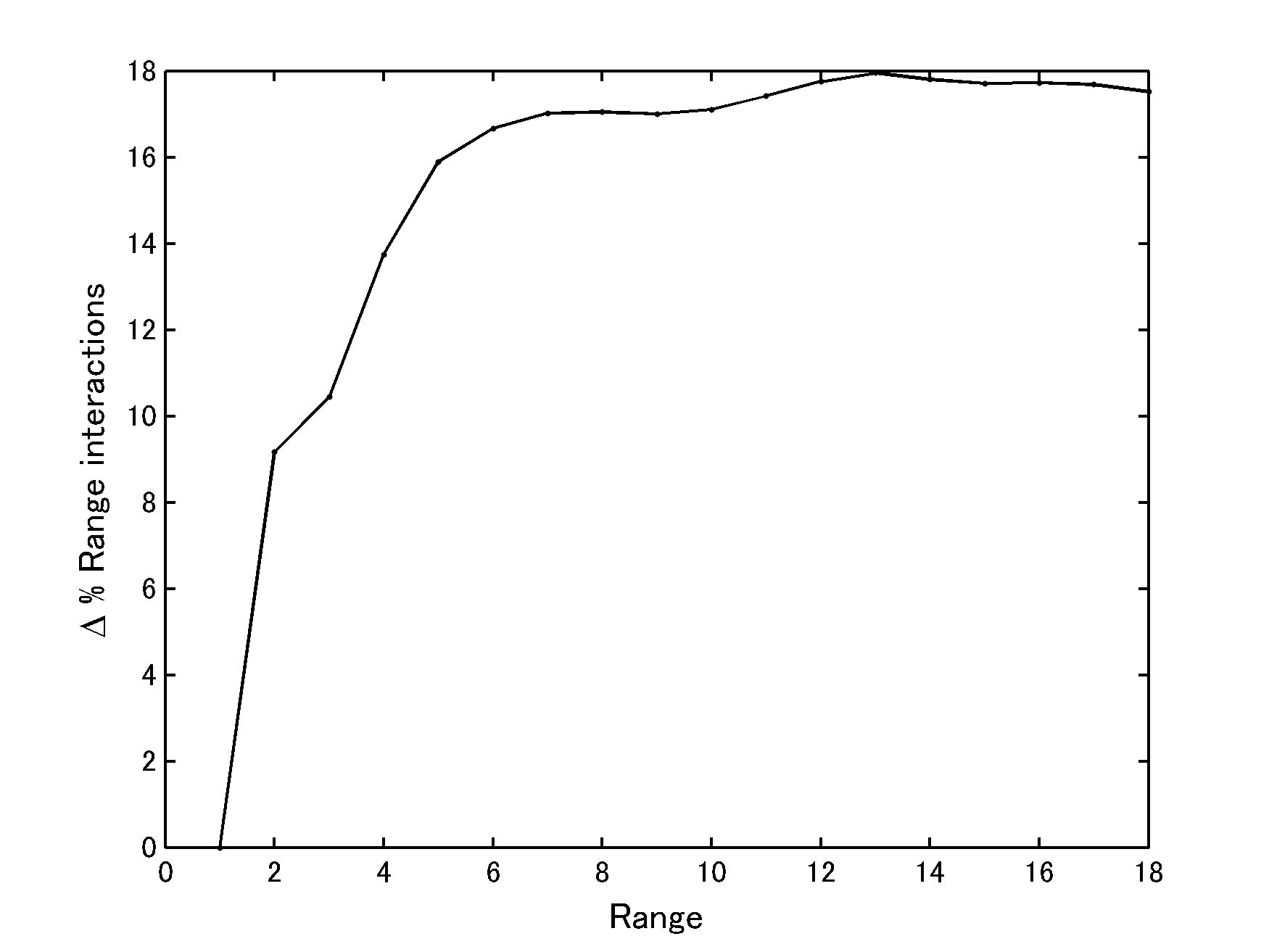


**Fig 3 Supp** Averages of the differences between the percentages of range interactions of the set of the intermodular residues minus the percentages of range interactions of the set of the intra-modular residues as a function of the range. The abscissa axis shows the range of the interactions. The ordinate axis was calculated using the equation: $\Delta\text{ \%Range interactions}=\left(\sum_{i}^{N} \text{(\% Range interactions)}_{_{i}}^{\text{B}}\text{-(\% Range interactions)}_{_{i}}^{\text{NB}}\right)/N$, where (%Range interactions) and (%Range interactions) are the percentages of long-range interactions for the sets of intermodular and intramodular residues respectively. *i* is the protein index in the multi-domain protein dataset.

**Table I Supp** Non-redundant dataset of 100 multi-domain proteins

| 10gsB | 12e8P | 13pkD | 1a02N | 1a04B | 1a0gA | 1a1fA | 1a1gA | 1a1hA | 1a1iA |
| --- | --- | --- | --- | --- | --- | --- | --- | --- | --- |
| 1a1jA | 1a1kA | 1a1lA | 1a1mA | 1a1nA | 1a1oA | 1a1rA | 1a21A | 1a22B | 1a2cH |
| 1a2oB | 1a31A | 1a35A | 1a3bH | 1a3eH | 1a3gB | 1a3qB | 1a3rH | 1a3wB | 1a3xB |
| 1a46H | 1a4jH | 1a4sA | 1a5aB | 1a5bB | 1a5dB | 1a5gH | 1a5kC | 1a5lC | 1a5mC |
| 1a5nC | 1a5oC | 1a61H | 1a6aA | 1a6tD | 1a71B | 1a7kD | 1a7lC | 1a81A | 1a8jL |
| 1a9bD | 1a9eA | 1aayA | 1abiH | 1abjH | 1abrB | 1abwA | 1abyA | 1acmD | 1acyH |
| 1ad0D | 1ad3A | 1ad5B | 1ad8H | 1ad9B | 1adbA | 1adcB | 1adeB | 1adiB | 1adqH |
| 1aduB | 1advB | 1ae6H | 1ae8H | 1af0A | 1af2A | 1afeH | 1afvK | 1agbA | 1agcA |
| 1agdA | 1ageA | 1agfA | 1agnB | 1agrD | 1agwA | 1aheB | 1ahfB | 1ahgB | 1ahtH |
| 1ahuB | 1ahvB | 1ahwF | 1ahxB | 1ahyA | 1ahzB | 1ai1H | 1ai4B | 1ai5B | 1ai6B |

**Table II Supp** Dataset of 115 proteins with conformers

| 2ran | 5croO | 1cewI | 3enl | 1ecbA | 4hvpA | 3icd | 6ldh | 1crl | 4mdhA |
| --- | --- | --- | --- | --- | --- | --- | --- | --- | --- |
| 1dqyA | 1sto | 1byuA | 1serA | 6timA | 1yptA | 9aatA | 8adh | 1j7nA | 1df0A |
| 1bu7A | 1coy | 1njgA | 1g59C | 1dv7A | 1wrpR | 1bncA | 2cblA | 1hnf | 1gtmA |
| 1ctr | 1gu0A | 1ddt | 1n0vC | 1erk | 1d9vA | 1jbvA | 1wdnA | 1e8bA | 1aonA |
| 1cu1A | 1lafE | 3mbp | 13pkA | 1qlnA | 1b7tA | 1mcpH | 2hmiA | 8atcA | 9gpbA |
| 1jmjA | 1e3iA | 1cbuB | 1bjyA | 2glsA | 1j74A | 1i69B | 2polA | 1pvuA | 1ex7A |
| 1jluE | 1quk | 1bpd | 2dri | 1prgA | 1k9pA | 1bjyA | 3tms | 1jmwA | 3chy |
| 1d5wA | 1cqrA | 1thv | 1lb4A | 4ctsA | 1g51B | 2eiaA | 3ezaA | 1i6iA | 1ba3 |
| 1jysA | 1ffh | 1evlA | 1i2dA | 4crxB | 1l5bA | 3dapA | 1jejA | 1dkxA | 1dppA |
| 2efgA | 2nacA | 1oxsC | 1ftoB | 1aa7A | 1ipd | 1lfg | 1g0xA | 1l96 | 1ejdA |
| 1rkm | 1gtrA | 1tde | 1fguA | 1bp5A | 1sspE | 1qf5A | 1d6mA | 7apiA | 1buyA |
| 1dkrA | 1g6oA | 1bam | 1q12A | 1pjr |  |  |  |  |  |

**Table III Supp** Functional site and fold centrally conserved residues (FCCRs) for the studied allosteric proteins. The functional site residues are indicated. The notations BS and Cat sites stand for Binding Site and Catalytic site, respectively. The information on sites marked with ^*^ was extracted from the reference indicated in the first column*.* *Dom* denotes those functional sites divided into several domains according to the CATH database.

| *Protein (ID)* | *Functional site residues* | *FCCR* |
| --- | --- | --- |
| *Hemoglobin (1bz0 A)*  Paoli et al, J Mol Biol.1996; Perutz et al, Annu Rev Biophys Biomol Struct*.* 1998; Suel et al, Nature Struct. Biol*.* 2002 | Hem BS:42,43,45,46,58,83,86,87,91,93,97,98,101  AB interface:27,30,31,34-36,103,104, 106,107,110, 111,113-115,117-120, 122,123,126 | 14,63,65,66,98,106,128 |
| *Glycogen phosphorylase (1e1y A)*  Johnson, FASEB J.1992; Oikonomakos et al,J.Biol.Chem 2000; Mitchell et al, Biochem 1996; | Cat site:568,569,574,676  AMP BS:67,71,72,75,76,309,310  280 loop^*^:281-286  Gly BS:397-437  Tower helix^*^:262-278 | 84,89,93,131,138,161,  295,297,490,568,608,648 |
| *Retinoic acid receptor RXR-alpha (1g5y A)*  Schulman A.I.et al,Cell 2004; Gampe R.T. et al ,Genes Dev 2000;Gampe R.T. et al, Mol Cell 2000 | Cat Ligand BS:265,268,269,271,272,275,305,306,309,310,312, 313,316,325-328,342,345,346,349,432,435,436,439  AF2 helix^*^:451,454,455  Coactivator BS:277,280,284,289,295,297,298,301,302,449,450, 453,454,456  AB interface:348,352,356,373,379,390,393,394,397,398,401, 415-417,419-424,426,427,430,431,434 | 300,305,309,310,315,371,376 |
| Catabolite gene activator protein (1g6n A) Busby S. et al, J.Mol.Biol 1999; Passner J.M. et al, J.Mol.Biol 2000; Harman J.G. Bichim.Biophys.Acta 2001; Fic E. et al, Biochem. 2006; Passner J.M. et al, PNAS 1997 | DNA BS:138,139,168-170,178-182,184,185,188,199  cAMP BS:49,61,62,70-73,82-84,86,127 | 63,64,65,69,123 |
| *Glutamate dehydrogenase (1hwz A)*  Smith T.J.et al, J.Mol.Biol 2001; Smith T.J. et al, J.Mol.Biol. 2002; Peterson E.P.et al, Structure 1999 | Cat site:126,168  NAPH BS:*Dom*A2:94,168-170 & *Dom*A3:215,250-255,275, 276,325-327,347-349,374,377  GTP BS: 209,210,213,217,261,262,265,289,292,450,454  Glutamate BS:*Dom*A2:90,92,111,114,126,166-168,199 & *Dom*A3:211,349, 378,381  Antenna^*^:391-445 | 90,110,173,211,252,347 |
| *Rhodopsine (1l9h A)*  Madabushi S. et al,J.Bio.Chem2004; Palczewski K. et al, Science 2000; Acharya S. et al, J.Biol.Chem. 1997; Ballesteros J.A. et al, Biochem. 2003; Brabazon D.M. et al, Biochem. 2003 | Retinal BS:113,114,117,118,121,122,186,187,189,191,207, 208,211,212,261,265,268,269,272,292,295,296  Gprot BS:71,72,148,226,230,250,253, 310-321 | 57,67,113,178,261,264, 265,268,293,301 |
| *Pyruvate kinase (1liu A)*  Valentini et al, J.Biol.Chem.2002; Munnoz et al, Comp Biochem Physiol B Biochem. Mol. Biol. 2003 | Cat site:*Dom*A2:116,313,371,405,407 & *Dom*A3:163  FBP BS:474-477,480,525,532,557,559,560-563,565  PEP BS:116,156,286,313,315,336,337,372,405,407 | 163,337,342,361,482,488 |
| *Phosphofructokinase (1pfk A)*  Fenton A.W. et al, Biochem 2003; Fenton A.W. et al, Biochem 2004; Lau et al, Biochem 1989 | Cat site:*Dom*A1:11,72,125,127 & *Dom*A2:171  FBP BS:*Dom*A1:11,72,125,127 & *Dom*A2:162,169-171,222, 243,249,252  MgADP BS:10,11,41,72,73,76,77,102-105,107,108 | 126,137,139,167,169 |
| *Tyrosine phosphatase 1B (1pty )*  Wiesmann C. et al, Nat Struct Mol Biol 2004; | Cat site:181,215,221,222  PhosphoTyr BS:46,182,215-221,262  Inhib BS:188,192,193,196,276,280 | 20,81,84,85,96,98,109, 124,194,199,214,254,257 |
| *Beta-trypsin (2ptc E)*  Hedstrom et al, Biochem*.* 1994; Suel et al, Nature Struct. Biol*.* 2002; Hung et al, Protein Eng. 1998; Szabo et al, J Mol Biol 2003 | Cat site:*Dom*A2:57,102 & *Dom*A1:193, 195,196,214  S1 site^*^:189-195,214-220,225-228  Loop1^*^:185-188  Loop2^*^:221,223-225  Loop3^*^:172-179 | 29,30,46,138,141,189, 194,212,213,228 |
| *G-protein s-alpha (1azs C)*  Buck et al, Sci STKE 2003; Chen et al, J.Biol.Chem. 2001; Grishina G. et al, J.Biol.Chem. 1997 | Cat site:*Dom*C1:50,201 & *Dom*C2:204, 227  GSP BS:*Dom*C1:48-55 & *Dom*C2:173,198-201,203,226,292, 293,295,296,365-367  Adenylyl cyclase BS ^*^:*Dom*C2:199-203 & *Dom*C1:204-216, 222-247,268-286 | 50,58,98,100,105,170, 173,176,201,265,293 |
| *G-protein beta-gamma (1tbg A)*  Buck et al, Sci STKE 2003; Buck et al, J.Biol.Chem. 2001 | PLC-Beta2 BS^*^:42-54, 86-105,117-135,228-249,321-340 | 18,22,61,63,105,150,151,190,192,234,252,258,278,289,318,320 |
| *Cytochrome P450eryF (1eup A)*  Cupp-Vickery et al,PNAS 2000 | Hem BS:90,91,98,102,109,238,241, 242,245,288,291,293,343-345,349,351,353,356,357  Andro1 BS:89,91,92,241,244,245,392  Andro2 BS:75,86,171,174,175,240,244, 391 | 102,238,249,289,293,344,349,358,393 |
